# Longitudinal in vivo imaging reveals asynchronous, incomplete Nipah virus clearance with prolonged focal CNS involvement in IFNAR^−/−^ mice

**DOI:** 10.1080/22221751.2026.2703394

**Published:** 2026-07-24

**Authors:** Katherine A. Davies, Stephen R. Welch, Joann D. Coleman-McCray, Georgia Ficarra, Jana M. Ritter, Teresa E. Sorvillo, Virginia Aida-Ficken, Shilpi Jain, César G. Albariño, Joel M. Montgomery, Michael K. Lo, Christina F. Spiropoulou, Jessica R. Spengler

**Affiliations:** a CDC Foundation assigned to Viral Special Pathogens Branch, Division of High-Consequence Pathogens and Pathology, Centers for Disease Control and Prevention, Atlanta, GA, USA; b Zoonotic and Emerging Disease Research Unit, National Bio and Agro-Defense Facility, Agricultural Research Service, United States Department of Agriculture, Manhattan, KS, USA; c Viral Special Pathogens Branch, Division of High-Consequence Pathogens and Pathology, Centers for Disease Control and Prevention, Atlanta, GA, USA; d Infectious Diseases Pathology Branch, Division of High-Consequence Pathogens and Pathology, Centers for Disease Control and Prevention, Atlanta, GA, USA; e Foreign Animal Disease Diagnostic Laboratory, National Veterinary Services Laboratories, National Bio and Agro-Defense Facility, United States Department of Agriculture, Manhattan, KS, USA

**Keywords:** Nipah virus, viral persistence, bioluminescence imaging, mice, central nervous system infections

## Abstract

Nipah virus (NiV) infection causes fatal acute respiratory and neurological disease. Survivors may develop long-term sequelae or experience relapsing encephalitis months to years after recovery. The basis of divergent disease trajectories and incomplete viral clearance remains poorly defined. Here, we establish a red-shifted bioluminescent resonance energy transfer reporter system to enable longitudinal tracking of NiV infection in vivo and apply it in mouse models of infection. We demonstrate route-, strain-, and immune-dependent differences in dissemination following NiV infection and reveal spatially heterogeneous and asynchronous viral clearance. While intranasal infection remains largely confined to the respiratory tract, intraperitoneal infection results in systemic spread followed by prolonged, fluctuating focal viral signal detectable up to 42 days post-infection in IFNAR^−/−^ mice. Integration with RT-qPCR, histopathology, and in situ hybridization showed persistent viral RNA in discrete brain regions after apparent clinical recovery, indicating anatomically restricted persistence. Together, these findings reveal the spatiotemporal dynamics of incomplete NiV clearance in vivo and provide a framework to study relapsing disease.

## Introduction

Nipah virus (NiV; *Paramyxoviridae*, *Henipavirus*) causes a broad clinical spectrum ranging from asymptomatic infection to severe respiratory and neurological illness [1]. Two genetically distinct strains are recognized: Nipah-Malaysia (NiV-M), which caused the first identified outbreak, and Nipah-Bangladesh (NiV-B), which has been responsible for recurrent outbreaks in India and Bangladesh since 2001 [1]. In some individuals, delayed-onset neurological complications, including relapsing encephalitis, develop months to years after apparent recovery (e.g. resolution of acute clinical signs) [2–4], and are hypothesized to result from incomplete viral clearance and persistence within anatomically restricted sites [4]. Supporting this possibility, viral antigen or RNA has been detected beyond conventional experimental endpoints (21–28 dpi) in surviving non-human primates (NHPs) and immunodeficient mice [5–7]. However, the spatiotemporal dynamics governing dissemination, tissue restriction, and clearance remain poorly defined.

Animal models recapitulate key respiratory and neurological features of human NiV infection and are essential for evaluating medical countermeasures [8]. Yet most studies rely on terminal or predefined early sampling timepoints, generating static snapshots of infection that obscure dynamic viral kinetics and inter-individual heterogeneity. Such approaches are particularly limiting for pathogens associated with relapse, where focal or fluctuating viral activity may persist in the absence of overt clinical signs.

Longitudinal, non-invasive imaging enables repeated whole-animal visualization within the same host, permitting direct assessment of viral dissemination and clearance over time. Prior studies in interferon (IFN) signalling-deficient mice demonstrated early, route-dependent spread following NiV-M infection [9], with later studies performed using recombinant derivatives of non-pathogenic Cedar virus (CeV) [10,11]. However, dynamic dissemination and potential persistence of pathogenic NiV strains beyond acute disease have not been directly visualized in vivo.

Here, we utilize a red-shifted bioluminescent resonance energy transfer (BRET) reporter system [12] to track NiV-M and NiV-B infection in immunocompetent and immunodeficient mice across different routes of exposure. In this system, a bioluminescent reporter is coupled to a fluorescent acceptor, producing red-shifted light emission following substrate administration. Compared with conventional bioluminescent reporters that emit blue-green light, red-shifted emission provides improved tissue penetration for in vivo imaging because it is less attenuated by absorption and scattering in host tissues. This approach supports sensitive detection of infection within deep tissues and visualization of viral dissemination from acute infection through convalescence. By integrating longitudinal imaging with molecular and histopathological analyses, we define route-, strain-, and immune-dependent dissemination patterns and identify prolonged, anatomically restricted reporter signal and viral RNA persistence. These findings establish a framework for investigating incomplete viral clearance and the biological processes associated with relapse.

## Methods

### Biosafety

Work with infectious viruses or infected animals was conducted in a biosafety level 4 (BSL-4) laboratory at the U.S. Centers for Disease Control and Prevention (CDC) following established BSL-4 standard operating procedures approved by the Institutional Biosafety Committee. Animal studies were approved by the CDC Institutional Animal Care and Use Committee (#3205). Work was performed in an AAALAC International-approved facility and conducted in accordance with the *Guide for the Care and Use of Laboratory Animals*. The CDC is fully accredited by AAALAC International.

### Viruses

Wild-type NiV-M (CDC VirHarv #813744, AF212302 [13]), obtained from a human cerebrum sample, was isolated in Vero E6 cells (ATCC-CRL-1586) and passaged once in Vero cells (African green monkey kidney; ATCC CCL-81). Wild-type NiV-B (CDC VirHarv #813747, GenBank AY988601 [14]), obtained from a human throat swab, was isolated in Vero E6 cells and passaged once in Vero cells. Recombinant NiV-M and NiV-B were engineered to express the bioluminescent red protein (BREP) construct [15], comprising a red-shifted NanoLuciferase (teLuc) linked to mScarlet-1 [12]. rNiV-M-BREP (CDC VirHarv #815142; GenBank PV753715.1) and rNiV-B-BREP (CDC VirHarv #815115; GenBank PV753716.1) were passaged three times in Vero cells. All viral stocks were verified by next-generation sequencing and confirmed to be mycoplasma-free.

### Mouse studies

Immunocompetent mice (C57BL/6J, 6 weeks old) were obtained from The Jackson Laboratory (Strain: 000664). Immunodeficient (IFNAR^−/−^) B6.129S2-1tm1Agt/Mmjax, RRID:MMRRC_032045-JAX mice (5–6 weeks old) were obtained from the Mutant Mouse Resource and Research Center (MMRRC) at The Jackson Laboratory, an NIH-funded strain repository, and were donated to the MMRRC by Michel Aguet, Ph.D., Swiss Institute for Experimental Cancer Research [16]. Mice were housed in a climate-controlled laboratory with a 12 h light/dark cycle and provided alfalfa-free commercial rodent chow (Teklad, Cat. no. 2018SC) and water *ad libitum*. Mice were group-housed in an isolator-caging system (Tecniplast, GM500 cages) with a HEPA-filtered inlet and exhaust air supply, on autoclaved corn cob bedding (Anderson Lab Bedding, Bed-o’Cobs ¼″) with cotton nestlets.

### Animal infection

Groups of mice (n = 4–8, male and female, evenly distributed) were infected intranasally (IN) or intraperitoneally (IP) with NiV-M, rNiV-M-BREP, NiV-B, or rNiV-B-BREP at a target dose of 10^6^ TCID_50_, or mock-infected IP with DMEM. Delivered dose was confirmed by titration of inoculum onto Vero cells (NiV-M: 1.7–1.9 × 10^6^ TCID_50_; rNiV-M-BREP: 1.4–2.0 × 10^6^ TCID_50_; NiV-B: 0.6–1.1 × 10^6^ TCID_50_; rNiV-B-BREP: 1.2–1.7 × 10^6^ TCID_50_). Body weight and clinical signs were assessed daily. Weight change was calculated relative to day 0 baseline values. Clinical scoring was assessed as described previously [17]. Mice were humanely euthanized by isoflurane exposure followed by cervical dislocation upon reaching euthanasia criteria, predefined study endpoints (28 days post-infection [dpi]), or study completion (42 dpi).

### In vivo imaging in mice

**Figure 1.**
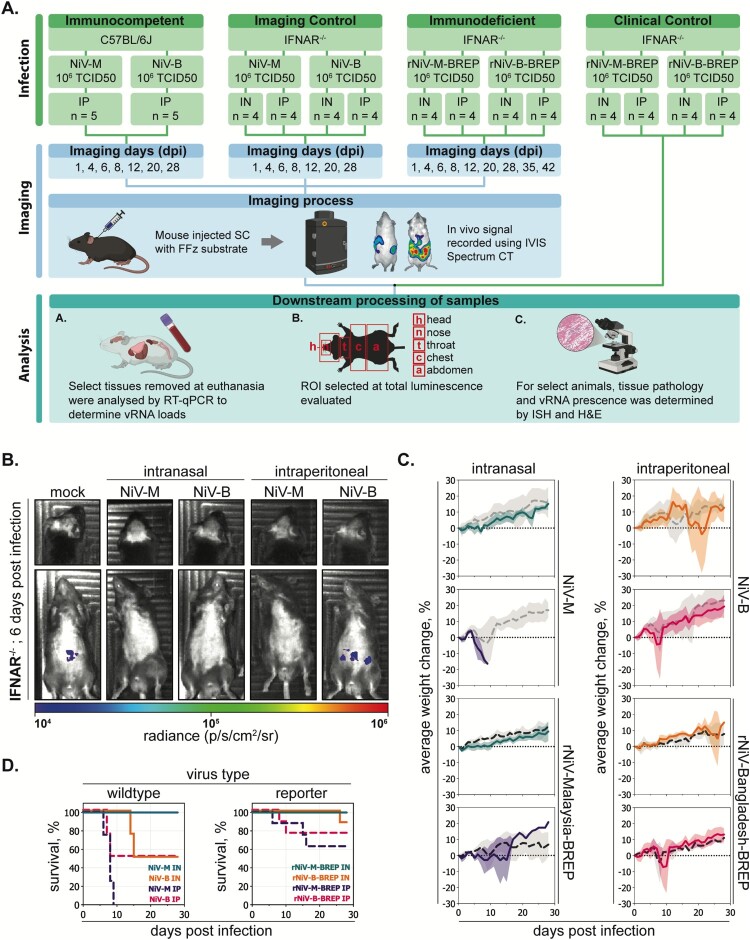
**Limited background and handling effects enable reliable longitudinal imaging of Nipah virus infection and dissemination. A.** In vivo imaging workflow. C57BL/6J or IFNAR^−/−^ mice were infected with Nipah virus Malaysia (NiV-M), recombinant NiV-M expressing BREP (rNiV-M-BREP), Nipah virus Bangladesh (NiV-B), or recombinant NiV-B expressing BREP (rNiV-B-BREP) intranasally (IN; IFNAR^−/−^ mice only) or intraperitoneally (IP) at 10^6^ TCID_50_. Mock-infected mice were included. A subset of mice was imaged using the IVIS Spectrum CT at 1, 4, 6, 8, 12, 16, 20, 28, 35, and 42 days post-infection (dpi) following administration of 0.75 µM fluorofurimazine (FFz). Signal intensity (total flux [p/s]) was quantified for each region of interest (ROI); dorsal (head [h]); ventral (nose [n], throat [t], chest [c], and abdomen [a]). At euthanasia, tissues, blood, and mucosal swabs were collected for viral RNA detection by RT-qPCR. Selected tissues were formalin fixed and analysed by in situ hybridization targeting NiV nucleoprotein mRNA/cRNA. **B.** Mock-infected, NiV-M-, or NiV-B-infected IFNAR**^−/−^** mice were imaged under identical conditions to those used for reporter-expressing NiV strains to assess background signal and artifact presence. Representative mice, at 6 dpi, are shown. Radiance (photons per second per square centimetre per steradian [p/s/cm^2^/sr]) is indicated by the scale bar. **C.** Mice were monitored daily for weight and clinical score. All non-reporter-infected and a subset of NiV-BREP-infected IFNAR^−/−^ mice were imaged. Weight change from baseline (day 0) is shown for imaged (colour) and non-imaged groups (dashed line, black). Historical controls (n = 18, dashed line, grey) are used as non-imaged comparators for non-reporter NiV strains. **D.** Kaplan-Meier survival curves for IFNAR**^−/−^** mice infected via the IN or IP route (dashed line) with NiV-M (n = 4/group), rNiV-Malaysia-BREP (n = 8/group), NiV-B (n = 4/group), or rNiV-B-BREP (n = 8/group).

Mice were imaged at predetermined time points (1, 4, 6, 8, 12, 16, 20, and 28 dpi; C57BL/6J and IFNAR^−/−^) selected based on previously observed acute and late-onset clinical signs [17], with additional later time points (35 and 42 dpi) included for IFNAR^−/−^ mice only (Figure 1(A), Table S1). To minimize signal interference, mice were shaved on the day of inoculation and as needed (∼2-week intervals). Mice were anaesthetized with isoflurane (2–2.5%) and injected subcutaneously (SC) in the caudal dorsum with 0.75 µM Nano-Glo® Fluorofurimazine In Vivo Substrate (FFz; Promega, Cat no. N4100) in a 200 µL volume. The injection site was selected to minimize injection-associated signal artifacts within regions of interest (ROIs). Following injection, mice were maintained under light anaesthesia (2–2.5%) for 5 min before transfer to the XIC-3 Animal Isolation Chamber (Revvity). Ventral and dorsal imaging was performed using the IVIS Spectrum CT system (Revvity), beginning 10 min post-substrate injection. Animals were maintained under light anaesthesia throughout imaging. Animals meeting euthanasia criteria on scheduled imaging days were imaged prior to euthanasia. Animals meeting euthanasia criteria between scheduled timepoints or found deceased were not imaged. Images were analysed using Living Image software (v4.8.2). BREP reporter signal, reflecting sites of protein expression and serving as an indirect marker of viral activity and spread in vivo, was quantified by calculating total flux (photons per second [p/s]) within ROIs defined for dorsal (head) and ventral (nose, throat, chest, and abdomen) aspects. ROIs were sized identically for each anatomical site across measurements.

### RNA extraction and real-time quantitative PCR

Small sections of liver, spleen, gonad (testes or ovary), kidney, heart, lung, eye, and brain were homogenized in 1 mL MagMAX™ Lysis/Binding Solution Concentrate (Thermo Fisher, Cat no. AM8500). Whole blood was collected into lithium heparin microtubes; 50 µL whole blood was added to 500 µL MagMAX Lysis/Binding Solution Concentrate. RNA was extracted from 250 µL lysate, with 150 µL isopropanol added at time of extraction, using the MagMAX Pathogen RNA/DNA kit (Thermo Fisher, Cat no. 4462359) on the KingFisher Apex System (Thermo Fisher). Samples were treated with DNase-1 (Biosearch Technologies, Cat no. D9905 K) and eluted in 75 µL elution buffer. Viral RNA (vRNA) was quantified using an RT-qPCR assay targeting the NiV nucleocapsid protein (N) sequence using the SuperScript III Platinum One-Step RT-qPCR kit (Thermo Fisher, Cat no. 11732088) [18]. Levels of vRNA were standardized using in-house RT-qPCR assays targeting the reference genes Peptidylprolyl isomerase A (*Ppia*) and Glucuronidase-beta (*Gusb*) [17]. Genome copies per µL extract were calculated against a standard curve of synthetic RNA diluted to known copy numbers. All primers, probes, and synthetic RNAs were synthesized by Integrated DNA Technologies (IDT).

### Histology and in situ hybridization

Representative sections of brain, heart, lung, liver, spleen, kidney, urinary bladder, pancreas, lymph nodes (submandibular, cervical, axillary, brachial, inguinal, pancreatic, and/or mesenteric), salivary glands, male or female reproductive tissues, and gastrointestinal tract, as available, were evaluated by histopathology. Histopathologic and in situ hybridization (ISH) analyses were performed on 9 of 12 mice surviving to 42 dpi, including five rNiV-M-BREP–infected mice (IN: 2 females, 1 male; IP: 1 female, 1 male) and four rNiV-B-BREP–infected mice (IN: 1 female, 1 male; IP: 1 female, 1 male). Necropsy tissues were fixed in 10% neutral buffered formalin for a minimum of 7 days and processed for routine paraffin histology. Sections were cut at 4 µm, mounted on glass slides, and stained with haematoxylin and eosin for histopathologic evaluation. Unstained tissue sections processed in the same way were used for ISH using RNAscope probes developed to detect N gene RNA (mRNA/cRNA) (Advanced Cell Diagnostics; V-Nipah-N-C, Cat no. 1193611-C1) according to the RNAscope 2.5 Assay protocol (ACD document nos. 322452 and 322360) to localize vRNA in tissues. Paraffin-embedded NiV-M-infected cells and paraffin-embedded NiV-B-infected brain tissue were used as positive controls.

### Graphing and statistical analysis

GraphPad Prism (v10) and R Statistical Software (v4) were used for statistical analysis and data visualization. Signal (total flux [p/s]) was log-transformed prior to analysis using a linear mixed-effects model to assess signal differences across distinct ROIs, comparing C57BL/6J and IFNAR^−/−^ mice, with signal as the dependent variable, group and time (dpi) as categorical fixed effects, and animal as a random effect to account for repeated measures. Type III analysis of variance with Satterthwaite’s method was performed on each ROI individually using the lmerTest package, with sum-to-zero contrasts applied. Signal differences from peak were assessed using the Kruskal–Wallis test with Dunn’s multiple comparisons test. Differences in daily weight change from baseline, comparing imaged and minimally handled groups, were assessed using unpaired Mann–Whitney tests with Holm–Šidák correction for multiple comparisons.

## Results

### BRET-based longitudinal imaging enables reliable tracking of Nipah virus infection in vivo

To ensure that longitudinal imaging reflected NiV infection rather than background or procedural artifacts, we established baseline signal characteristics and assessed potential confounders associated with reporter expression, repeated handling, and anaesthesia. IFNAR**^−/−^** mice were mock-infected or infected with wild-type (non-reporter-expressing) NiV strains and imaged in parallel with rNiV-M-BREP and rNiV-B-BREP cohorts at all time points through 28 dpi (Figure 1(A), Figures S1 and S2). Mock- and non-reporter-virus-infected mice exhibited low-level injection site signal (4 × 10^3^–3 × 10^5^ p/s) and sporadic abdominal background (<3 × 10^5^ p/s; Figure 1(B)). To distinguish reporter-associated signal from non-specific background, the mean signal for each ROI was calculated from mock-infected animals and used as a region-specific threshold for interpreting signal in rNiV-BREP-infected animals. Similar low-level signals were observed in mock- and wild-type NiV-infected mice, without sustained or region-specific signal increases associated with non-reporter virus infection.

We next evaluated whether repeated anaesthesia and imaging influenced disease progression. Imaged cohorts were compared with minimally handled mice for both wild-type NiV-infected and rNiV-BREP-infected groups. No statistically significant differences in weight trajectories were observed, and disease manifestations and kinetics were broadly comparable between imaged and non-imaged mice through 28 dpi (Figure 1(C)). However, rNiV-BREP infection was mildly attenuated relative to non-reporter virus infection (Figure 1(D)). Together, these controls support the use of BREP-based imaging to resolve the spatiotemporal dynamics of NiV dissemination in vivo.

### Longitudinal imaging reveals transient and self-limited Nipah virus replication in immunocompetent mice

**Figure 2.**
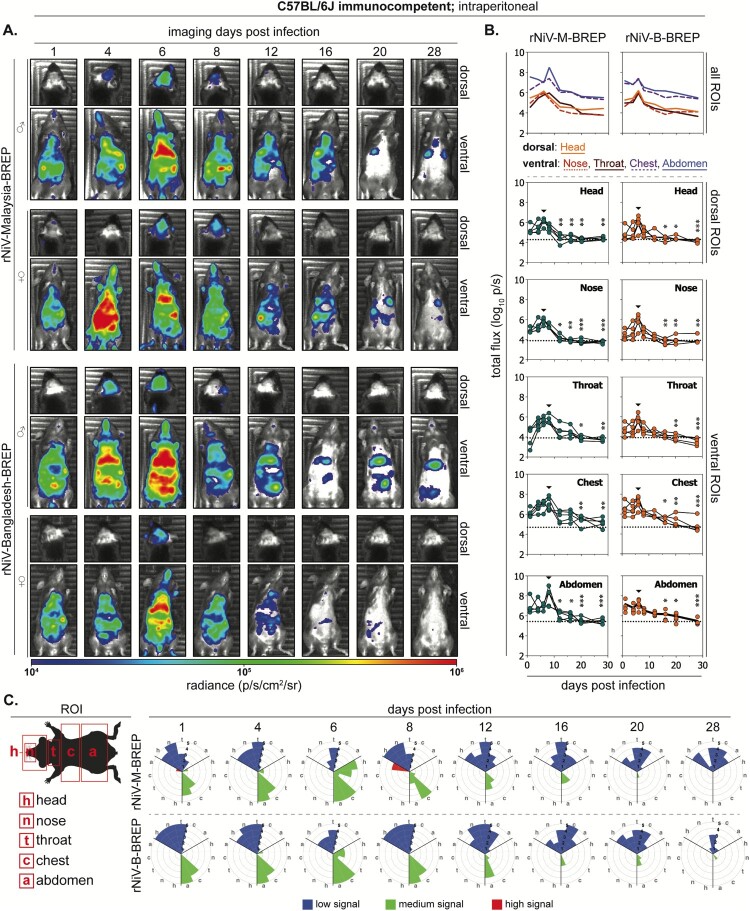
**Widespread dissemination of Nipah virus in immunocompetent mice following intraperitoneal infection. A.** C57BL/6J mice (n = 5/group) were infected intraperitoneally with 10^6^ TCID_50_ recombinant NiV-Malaysia expressing BREP (rNiV-Malaysia-BREP) or recombinant NiV-Bangladesh expressing BREP (rNiV-Bangladesh-BREP) and imaged at 1, 4, 6, 8, 12, 16, 20, and 28 days post-infection (dpi). Representative male (♂) and female (♀) mice are shown. Radiance (photons per second per square centimetre per steradian [p/s/cm^2^/sr]) is indicated by the scale bar. **B.** Signal intensity (total flux [p/s]) was quantified for each region of interest (ROI); dorsal (head) and ventral (nose, throat, chest, and abdomen). Top panels show group means for each ROI (head [solid light orange line], nose [dashed dark orange line], throat [solid red line], chest [dashed purple line], and abdomen [solid lilac line]). Lower panels indicate individual animal signal trajectories. ROI-specific baselines (dotted line) were calculated from mock-infected mice across all imaging timepoints (1, 4, 6, 8, 12, 16, 20, and 28 dpi); dorsal – head: 2 × 10^4^ p/s, ventral – nose: 8 × 10^3^ p/s, throat: 4 × 10^3^ p/s, chest: 5 × 10^4^ p/s, and abdomen: 3 × 10^5^ p/s. Statistical differences relative to peak (▾) were assessed using the Kruskal-Wallis test with Dunn’s multiple comparisons test (***p ≤ 0.001; **p ≤ 0.01; *p ≤ 0.05). Non-significant comparisons are not shown. **C.** Radar plots indicate the number of animals with low (baseline–10^6^ p/s; blue), medium (10^6^–10^8^ p/s; green), or high (>10^8^ p/s; red) signal intensity at each ROI; animals with signal below ROI-specific baselines were not included. Signal classifications are based on quantified signal rather than visual assessment.

Immunocompetent mice support NiV replication in the absence of clinical signs or lethal disease [19,20]. To define the spatiotemporal dynamics of infection under intact antiviral immunity and establish a baseline for comparison with immunodeficient mice, C57BL/6J mice were infected IP with rNiV-M-BREP or rNiV-B-BREP (n = 5/group) and imaged through 28 dpi (Figure 1(A)). No clinical signs were observed throughout the study period (Table S1). Reporter signal was detectable by 1 dpi, predominantly in the chest and abdomen. At 4 dpi, signal intensified in the chest and became apparent in the head (Figure 2(A), Figures S3 and S4). Peak signal across all regions was observed at 6–8 dpi (Figure 2(B)). Signal declined progressively after peak in a region-specific manner: head-associated signal decreased most rapidly, reaching near-background levels by 12–16 dpi (significant reduction from peak, *p* < 0.05), whereas chest and abdominal signal declined more gradually, with significant reductions from peak observed by 16–20 dpi and 8–16 dpi, respectively (*p* < 0.05; Figure 2(B)).

During resolution (8–12 dpi), signal frequently consolidated into discrete focal regions before diminishing. By 28 dpi, reporter signal had returned to background levels in most mice. However, low-level (<10^6^ p/s) focal signal in the chest persisted in a subset of mice, more frequently following rNiV-M-BREP infection (4/5 [80%]) than rNiV-B-BREP infection (2/5 [40%]; Figure 2(C)). Despite these residual signals, no vRNA was detected in any tissue by RT-qPCR at study endpoint (28 dpi).

### Absence of type I interferon signalling permits widespread dissemination and altered clearance kinetics

**Figure 3.**
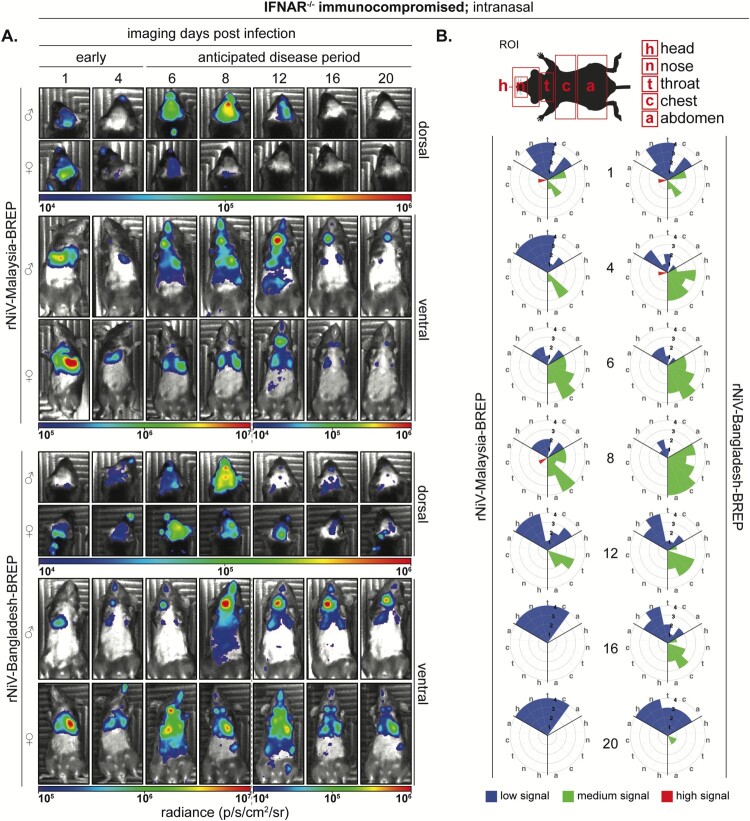
**Intranasal Nipah virus infection of IFNAR^−/−^ mice results in upper-body dissemination.** IFNAR**^−/−^** mice were infected intranasally with 10^6^ TCID_50_ recombinant NiV-Malaysia expressing BREP (rNiV-Malaysia-BREP) or recombinant NiV-Bangladesh expressing BREP (rNiV-Bangladesh-BREP) and imaged (n = 4/group) at early, preclinical (1 and 4 days post-infection [dpi]) and anticipated clinical disease (6, 8, 12, 16, and 20 dpi). **A.** Representative images of male (♂) and female (♀) mice are shown. Radiance (photons per second per square centimetre per steradian [p/s/cm^2^/sr]) is indicated by the scale bar. The dashed vertical line between 8 and 12 dpi imaging panels indicate a change in radiance scale. **B.** Signal intensity (total flux [p/s]) was quantified for each region of interest (ROI); dorsal (head) and ventral (nose, throat, chest, and abdomen). Radar plots indicate the number of animals with low (baseline–10^6^ p/s; blue), medium (10^6^–10^8^ p/s; green), or high (>10^8^ p/s; red) signal intensity at each ROI. ROI-specific baselines (dotted line) were calculated from mock-infected mice across all imaging timepoints (1, 4, 6, 8, 12, 16, 20, and 28 dpi); dorsal – head: 2 × 10^4^ p/s, ventral – nose: 8 × 10^3^ p/s, throat: 4 × 10^3^ p/s, chest: 5 × 10^4^ p/s, and abdomen: 3 × 10^5^ p/s. Animals with signal below ROI-specific baselines were not included. Signal classifications are based on quantified signal rather than visual assessment.

**Figure 4.**
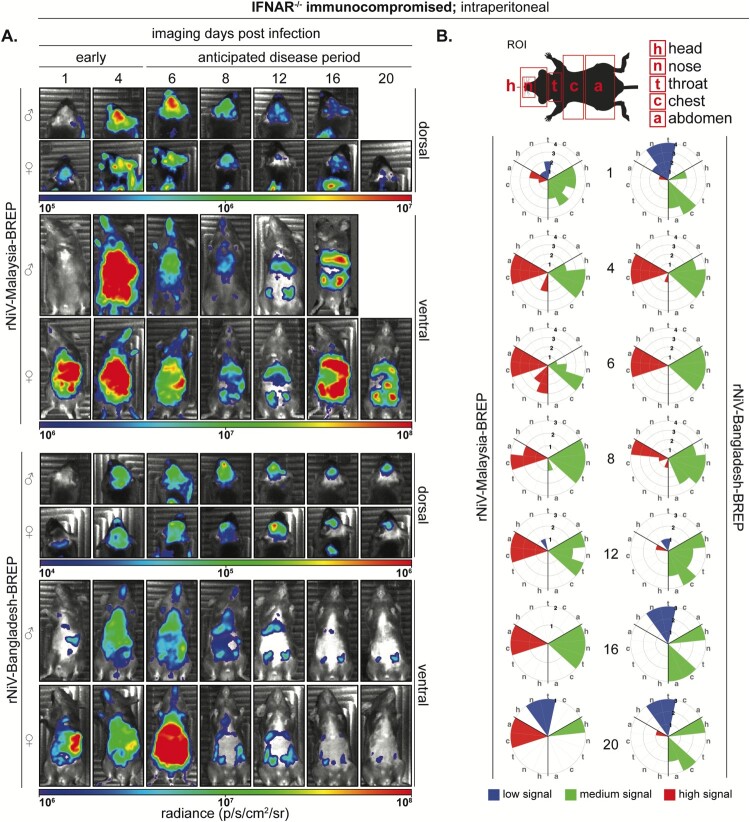
**Intraperitoneal Nipah virus infection of IFNAR^−/−^ mice results in widespread dissemination and sites of persistent reporter-associated signal.** IFNAR**^−/−^** mice were infected intraperitoneally with 10^6^ TCID_50_ recombinant NiV-Malaysia expressing BREP (rNiV-Malaysia-BREP) or recombinant NiV-Bangladesh expressing BREP (rNiV-Bangladesh-BREP) and imaged (n = 4/group) at early, preclinical (1 and 4 days post-infection [dpi]) and anticipated clinical disease (6, 8, 12, 16, and 20 dpi). **A.** Representative images of 1 male (♂) and 1 female (♀) are shown. Radiance (photons per second per square centimetre per steradian [p/s/cm²/sr]) is indicated by the scale bar. **B.** Signal intensity (total flux [p/s]) was quantified for each region of interest (ROI); dorsal (head) and ventral (nose, throat, chest, and abdomen). Radar plots indicate the number of animals with low (baseline–10^6^ p/s; blue), medium (10^6^–10^8^ p/s; green), or high (>10^8^ p/s; red) signal intensity at each ROI. ROI-specific baselines (dotted line) were calculated from mock-infected mice across all imaging timepoints (1, 4, 6, 8, 12, 16, 20, and 28 dpi); dorsal – head: 2 × 10^4^ p/s, ventral – nose: 8 × 10^3^ p/s, throat: 4 × 10^3^ p/s, chest: 5 × 10^4^ p/s, and abdomen: 3 × 10^5^ p/s. Animals with signal below ROI-specific baselines were not included. Signal classifications are based on quantified signal rather than visual assessment.

We next defined how impaired antiviral signalling influences NiV dissemination by longitudinally imaging IFNAR^−/−^ mice infected IN or IP with rNiV-M-BREP (Figure 3(A)) or rNiV-B-BREP (Figure 4(A)). Animals were imaged during early infection (1 and 4 dpi) and anticipated disease period (6–20 dpi) [17], with signal quantified across head, nose, throat, chest, and abdominal regions (Figure 1(A)).

**Figure 5.**
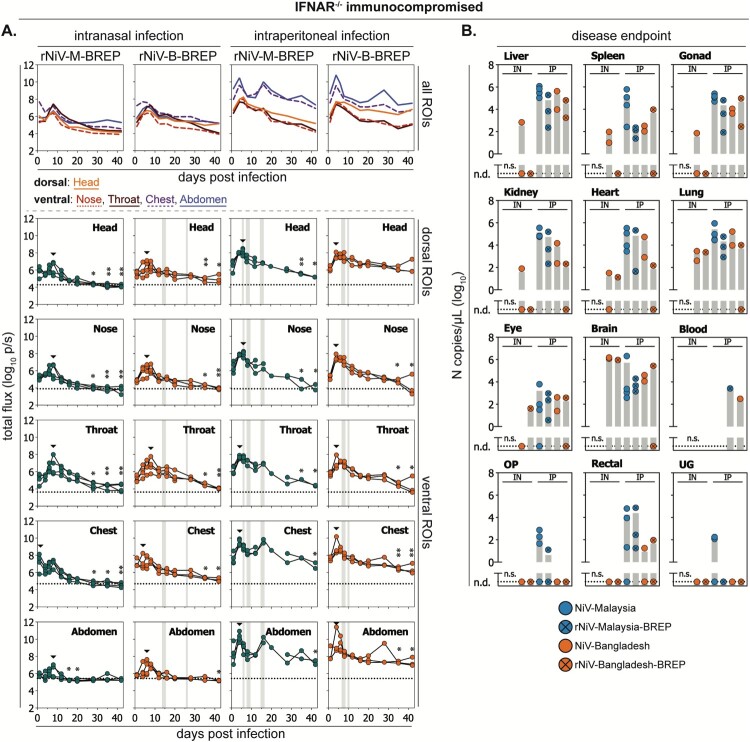
**Distinct region-specific patterns of Nipah virus dissemination and clearance in IFNAR^−/−^ mice**. IFNAR**^−/−^** mice were infected intranasally or intraperitoneally with 10^6^ TCID_50_ recombinant NiV-Malaysia expressing BREP (rNiV-Malaysia-BREP) or recombinant NiV-Bangladesh expressing BREP (rNiV-Bangladesh-BREP) and imaged (n = 1–4/group) at 1, 4, 6, 8, 12, 16, 20, 28, 35, and 42 days post-infection (dpi). **A.** Signal intensity (total flux [p/s]) was quantified for each region of interest (ROI); dorsal (head) and ventral (nose, throat, chest, and abdomen). Top panels show group means for each ROI (head [solid light orange line], nose [dashed dark orange line], throat [solid red line], chest [dashed purple line], and abdomen [solid lilac line]). Lower panels indicate individual animal signal trajectories. Grey shading indicates animals reaching endpoint criteria, including both reporter-expressing and wild-type NiV strains. ROI-specific baselines (dotted line) were calculated from mock-infected mice across all imaging timepoints (1, 4, 6, 8, 12, 16, 20, and 28 dpi); dorsal – head: 2 × 10^4^ p/s, ventral – nose: 8 × 10^3^ p/s, throat: 4 × 10^3^ p/s, chest: 5 × 10^4^ p/s, and abdomen: 3 × 10^5^ p/s. Statistical differences relative to peak (▾) were calculated using the Kruskal-Wallis test with Dunn’s multiple comparisons test (***p ≤ 0.001; **p ≤ 0.01; *p ≤ 0.05). Non-significant comparisons are not shown. **B.** Tissue (liver, spleen, gonad [testis, ovary], kidney, heart, lung, eye, and brain), blood, and mucosal swabs (oropharyngeal, rectal, and urogenital) were collected from animals reaching terminal endpoints. RNA was extracted and viral RNA loads (nucleoprotein [N] gene copies per µL) were quantified. Each data point represents an individual animal, the grey bar indicates the group mean. “n.d.” no RNA was detected, “n.s.” no samples were available.

Following IN infection, reporter signal was detectable by 1 dpi, predominantly in the chest in all mice (Figure 3(A), Figure S5). Low-level head involvement was observed in most mice (<10^6^ p/s), with moderate head signal evident in a subset of rNiV-M-BREP-infected mice (2/4 at 10^6^–10^8^ p/s; Figure 3(B)). By 4 dpi, chest and head signal declined in most animals, while focal throat involvement emerged in 25% of rNiV-M-BREP-infected mice and 50% of rNiV-B-BREP-infected mice. Across both strains, chest-associated signal peaked during early infection (1–4 dpi), whereas head, throat, and abdominal signal reached maximal intensity by 6–8 dpi (Figure 5(A)). Thereafter, signal declined progressively across all regions through 20 dpi, with signal consolidating into discrete focal regions within the throat in all IN-infected animals over time (Figure 3(A)). These data demonstrate that, in the absence of type I interferon signalling, IN exposure produces early respiratory tissue-associated replication followed by dissemination to additional anatomical sites prior to eventual signal reduction.

In contrast, IP infection resulted in rapid and widespread dissemination. Reporter signal, evident by 1 dpi, initially concentrated in the abdomen (∼10^7^–10^9^ p/s; Figure 4(A), Figure S6), and expanded by 4 dpi to involve all anatomical regions. Peak signal intensity was reached at 4–6 dpi across head (∼10^7^–10^8^ p/s), chest (∼10^8^–10^10^ p/s), and abdominal (∼10^8^–10^11^ p/s) regions, reflecting systemic viral spread (Figure 5(A)). Notably, elevated chest or head signal preceded the onset of overt respiratory (rNiV-M-BREP, n = 3) or neurological (rNiV-B-BREP, n = 1) signs, respectively, suggesting that longitudinal imaging detects pathogenic dissemination prior to terminal clinical deterioration. At terminal endpoints, high vRNA levels were detected across multiple tissues by RT-qPCR in both reporter-expressing and non-reporter NiV infections (Figure 5(B)).

Dissemination kinetics differed by viral strain. Following IP rNiV-M-BREP infection, a secondary increase in signal was observed in the chest and abdominal regions at 16 dpi (Figure 5(A)), at levels comparable to initial peak. In contrast, rNiV-B-BREP infection demonstrated a more progressive decline across head, nose, throat, and chest regions; however, residual moderate-to-high signal persisted in the head, chest, and abdomen of surviving animals throughout the study period (10^6^–10^8^ to >10^8^ p/s; Figure 4(B)).

**Figure 6.**
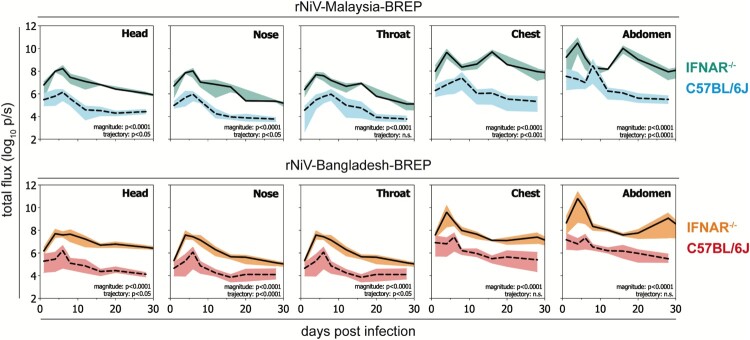
**Comparison of Nipah virus-infected immunocompetent and immunodeficient mice reveals background-dependent differences in dissemination and clearance kinetics.** C57BL/6J mice (n = 5/group, dotted lines) and IFNAR^−/−^ mice (n = 1–4/group, solid lines) were infected intraperitoneally with 10^6^ TCID_50_ recombinant NiV-M expressing BREP (rNiV-Malaysia-BREP) or recombinant NiV-B expressing BREP (rNiV-Bangladesh-BREP) and imaged at 1, 4, 6, 8, 12, 16, 20, and 28 days post infection. Signal intensity (total flux [p/s]) was calculated for each region of interest (ROI); dorsal (head); ventral (nose, throat, chest, and abdomen). Black lines indicate group mean, with the shaded area representing the range. Statistical differences between groups were assessed using a linear mixed-effects model, with type III analysis of variance performed using Satterthwaite’s method.

Direct comparison between host backgrounds revealed significantly greater signal following IP infection of IFNAR^−/−^ mice relative to C57BL/6J mice across all anatomical regions (*p* < 0.0001; Figure 6). Temporal signal progression in the head differed significantly (*p* < 0.05) between IFNAR^−^/^−^ and immunocompetent mice for both NiV strains. Significant differences in temporal signal were also observed in the chest (*p* < 0.001) and abdomen (*p* < 0.0001) with rNiV-M-BREP.

Together, these findings demonstrate that the intact type I IFN response constrains the magnitude and temporal progression of NiV dissemination, whereas loss of IFN signalling results in rapid systemic spread, strain-dependent amplification patterns, and delayed clearance kinetics. Longitudinal imaging further reveals that dissemination dynamics precede clinical manifestations, underscoring the utility of real-time visualization for elucidating pathogenesis beyond terminal endpoints.

### Incomplete viral clearance and anatomically restricted persistence following apparent recovery

To assess persistent reporter signal beyond acute disease, surviving IFNAR^−/−^ mice infected with rNiV-M-BREP or rNiV-B-BREP were imaged at the conventional study endpoint (28 dpi) and at extended convalescent intervals (35 and 42 dpi). Animals were selected to ensure representation of both sexes and infection routes.

**Figure 7.**
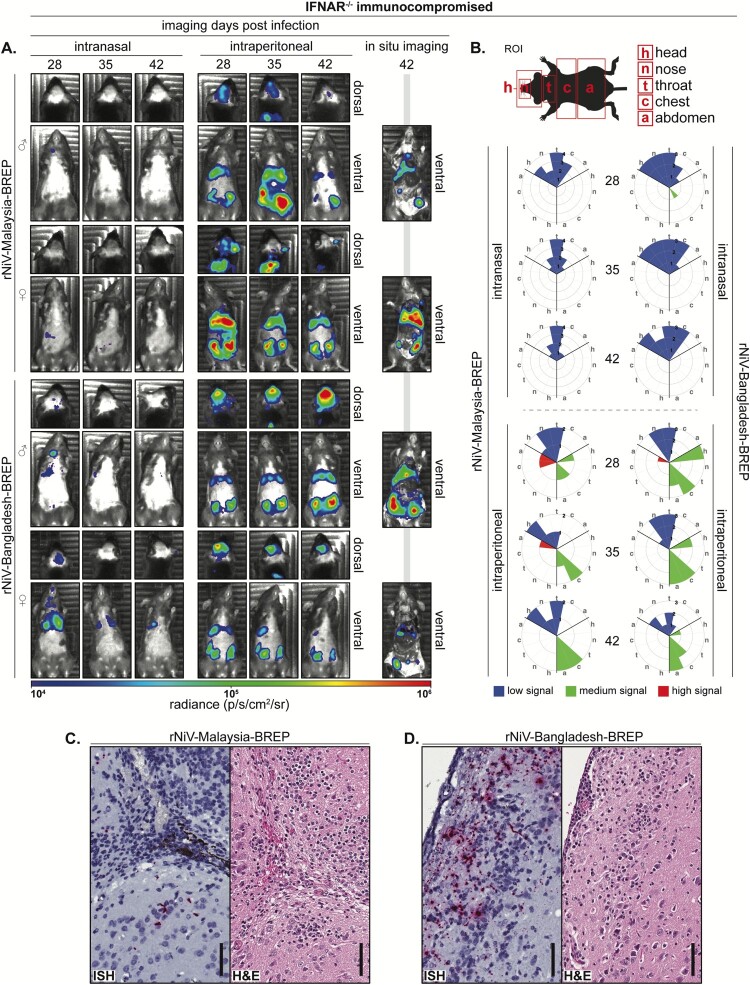
In vivo imaging and in situ hybridization identify sites of reporter signal and RNA persistence in IFNAR^−/−^ mice after apparent clinical recovery at 42 dpi. A. IFNAR^−/−^ mice were infected intranasally or intraperitoneally with 10^6^ TCID_50_ recombinant NiV-Malaysia expressing BREP (rNiV-Malaysia-BREP) or recombinant NiV-Bangladesh expressing BREP (rNiV-Bangladesh-BREP) and imaged during convalescence (28, 35, and 42 days post-infection; n = 2–4/group). One previously non-imaged rNiV-Malaysia-BREP IP-infected mouse was included to ensure representation of both sexes. Representative images of male (♂) and female (♀) mice are shown. Radiance (photons per second per square centimetre per steradian [p/s/cm^2^/sr]) is indicated by the scale bar. B. Signal intensity (total flux [p/s]) was quantified for each region of interest (ROI); dorsal (head) and ventral (nose, throat, chest, and abdomen). Radar plots indicate the number of animals with low (baseline–10^6^ p/s; blue), medium (10^6^–10^8^ p/s; green), or high (>10^8^ p/s; red) signal intensity at each ROI. ROI-specific baselines (dotted line) were calculated from mock-infected mice across all imaging timepoints (1, 4, 6, 8, 12, 16, 20, and 28 dpi); dorsal – head: 2 × 10^4^ p/s, ventral – nose: 8 × 10^3^ p/s, throat: 4 × 10^3^ p/s, chest: 5 × 10^4^ p/s, and abdomen: 3 × 10^5^ p/s. Animals with signal below ROI-specific baselines were not included. Signal classifications are based on quantified signal rather than visual assessment. C. Brain from an rNiV-Malaysia-BREP inoculated mouse with rare granular in situ hybridization (ISH) staining in the ventral forebrain. Haematoxylin and eosin (H&E)-stained section of brain shows mild congestion and gliosis in the area with ISH staining. D. Brain from a rNiV-Bangladesh-BREP-inoculated mouse showing focally extensive ISH staining in the dorsal cerebral cortex and leptomeninges. H&E-staining shows inflammatory changes, with glial proliferation and satellitosis, and neuronal degeneration. C, D. Original magnifications, ×40; scale bar, 60 µm.

Despite survival beyond the acute phase, measurable signal was present at 28 dpi in multiple animals in the chest and throat (Figure 7(A and B)). Following IN infection, signal resolution occurred more gradually with rNiV-B-BREP infection. Significant reductions from peak (*p* < 0.05) were not observed until 35 dpi, compared with 28 dpi following rNiV-M-BREP infection (Figure 5(A)). Signal across all anatomical regions approached baseline levels by 42 dpi.

In contrast, IP infection resulted in durable and anatomically widespread signal persistence. At 28 dpi, reporter signal was present in the head (>10^5^ p/s), chest (10^6^–10^8^ p/s), and abdomen (10^6^–10^8^ p/s) of surviving animals (Figure 7(A and B)). In rNiV-B-BREP-infected mice, head-associated signal frequently localized to a discrete central focus. Signal declined progressively in IP-infected animals but remained detectable through 42 dpi. Interindividual variability was observed; focal increases in abdominal signal were observed in specific individuals at 28 dpi (rNiV-B-BREP) and 35 dpi (rNiV-M-BREP), and renewed signal intensification in the head and abdomen of another at 42 dpi (rNiV-B-BREP), indicating fluctuating rather than uniformly resolving reporter signal. Post-euthanasia, in situ imaging of IP-infected mice did not attribute thoracic or abdominal signal to a single dominant organ source (Figure 7(A)), suggesting either multifocal or spatially restricted signal not confined to a identifiable structure.

To determine whether persistent imaging signal corresponded to vRNA, RT-qPCR was performed on multiple tissues collected from all surviving animals at 28 dpi (NiV-M, NiV-B, rNiV-M-BREP, rNiV-B-BREP) and 42 dpi (rNiV-M-BREP, rNiV-B-BREP). Most tissues were negative or contained low-level vRNA (<10³ N copies/µL). However, one rNiV-B-BREP-infected mouse, demonstrating increased head and abdominal signal at 42 dpi, exhibited markedly elevated vRNA in the brain (1.6 × 10⁶ N copies/µL) (Figure S7), despite apparent clinical recovery.

Collectively, these findings demonstrate that in the absence of intact type I IFN signalling, NiV infection may fail to achieve uniform viral clearance following apparent recovery (e.g. resolution of acute clinical signs). Longitudinal imaging reveals prolonged, anatomically restricted, and, in some cases fluctuating viral signal during convalescence, particularly following intraperitoneal exposure. These data provide in vivo evidence for compartmentalized viral signal following apparent recovery and establish a framework for interrogating mechanisms underlying persistence and relapse.

### Brain-restricted viral RNA persistence confirmed by in situ hybridization and histopathology

To determine whether late-stage imaging signal reflected viral persistence, and to define accompanying pathological changes, tissues from IFNAR^−/−^ mice surviving to 42 dpi (n = 9) were examined by histopathology and in situ hybridization (ISH) using a probe targeting NiV N gene mRNA/cRNA. Animals were prioritized for analysis based on persistent reporter signal and/or detectable vRNA at late time points to maximize sensitivity for detecting residual infection.

Detection of NiV RNA by ISH was not universal; vRNA was detected exclusively in the brains of two IP-infected mice (one rNiV-M-BREP and one rNiV-B-BREP). In the rNiV-M-BREP-infected mouse, rare focal ISH staining localized to a small region of the olfactory peduncle and ventral forebrain (Figure 7(C)); staining corresponded to a focus of mild vascular congestion and gliosis. In the rNiV-B-BREP–infected mouse, more extensive ISH signal was observed (Figure 7(D)), localizing to a linear region of the outer, dorsal cerebral cortex and leptomeninges, which had lymphoplasmacytic inflammation, glial proliferation and satellitosis, and neuronal degeneration. Notably, this same animal had high levels of BREP signal and vRNA in the brain at 42 dpi. In both animals, staining was granular and present both throughout the neuropil and in close association with cellular nuclei but could not be localized to any specific cell type; however, no apparent endothelial staining was seen in either animal. vRNA was not detected by ISH in these two animals outside the brain or in any tissue from other animals. One animal had a large leptomeningeal vessel with mural inflammation but no ISH staining. No histopathologic changes attributable to NiV infection were identified in other tissues.

These findings provide anatomical confirmation of focal, brain-restricted vRNA persistence following apparent clinical recovery. The restriction of vRNA to discrete brain foci, in the absence of systemic involvement, supports the concept of anatomically confined, incomplete viral clearance and is consistent with reports of late-onset and relapsing encephalitis following human infection.

## Discussion

Relapsing encephalitis and delayed-onset neurological disease following NiV infection have long implied that viral clearance may be incomplete in a subset of individuals. Persistence has largely been inferred from terminal sampling and cross-sectional tissue analyses. Here, longitudinal in vivo imaging enabled real-time visualization of NiV infection in both immunocompetent and immunodeficient mice, providing a dynamic view of viral dissemination and resolution. Longitudinal imaging revealed spatially heterogeneous and asynchronous clearance, characterized by signal confined to discrete regions, secondary increases in intensity, and fluctuating patterns during convalescence. Unlike traditional molecular and histopathological approaches, which are limited to selected tissues or sections, whole-animal imaging can identify unexpected or spatially restricted sites of infection. In this study, reporter signal detected at late time points guided tissue selection for histopathological analysis, which identified focal vRNA localization within the brain of IFNAR^−/−^ mice. Prior work using a rNiV-ZsGreen1 reporter similarly demonstrated focal signal within the brain [21]. Together, these findings suggest that conventional tissue sampling approaches may underestimate residual infection at later time points particularly when viral persistence is focal rather than broadly disseminated.

Consistent with prior studies, immunocompetent mice supported NiV infection but remained subclinical, whereas IFNAR^−/−^ mice developed widespread viral dissemination and severe disease [7,17,20,22]. Our findings extend this paradigm by demonstrating that, in immunocompetent mice, signal intensity was lower and resolution occurred more rapidly across tissues, reinforcing a central role for the type I IFN response in restricting the magnitude and kinetics of NiV replication. In contrast, IFNAR^−/−^ mice exhibited systemic amplification, delayed resolution, and focal convalescent signal. These data suggest that IFN signalling promotes spatially coordinated viral clearance and limits prolonged persistence of reporter signal and vRNA within focal tissue sites. Similar IFN-dependent restriction of systemic dissemination and viral burden has been observed in other viral models, including West Nile [23], Dengue [24], Zika [25], and Japanese Encephalitis viruses [26].

Evidence from both animal models and human cases further supports the possibility of prolonged CNS infection following apparent recovery. Detection of vRNA in the brains of surviving NHPs [5], Syrian hamsters [27], and IFNAR^−/−^ mice [7] has been reported weeks after infection, even in the absence of overt clinical disease. In addition, the detection of viral antigen in neurons following delayed-onset NiV encephalitis in humans provides evidence for incomplete viral clearance within immune-privileged sites months to years after recovery [4]. However, infectious NiV has not yet been recovered from relapse cases or persistent animal models. These reports, along with persistent reporter signal, RT-qPCR detection of brain RNA, and ISH localization of vRNA to discrete cortical and forebrain regions, provide biological evidence consistent with incomplete viral clearance from the CNS following recovery. Neuronal persistence is not unique to NiV; measles virus, a related paramyxovirus, can persist in CNS neurons and lymphoid tissues [28], illustrating that neurotropic paramyxoviruses may evade immune clearance within restricted compartments. These findings support a model of incomplete NiV clearance in discrete CNS niches following apparent recovery. However, the presence of replication-competent virus cannot be determined from the current data and would require isolation of infectious virus from affected tissues. This may be challenging at late time points due to low viral burden and potential interference from neutralizing antibodies or sample toxicity.

Distinct dissemination trajectories were observed following IN versus IP infection, indicating that route of exposure influences early spread and subsequent tissue involvement. Following IN inoculation, signal localized predominantly to the chest early after infection, consistent with confinement to the respiratory tract. In contrast, IP inoculation led to rapid systemic dissemination, reflecting early access to systemic compartments. Head-associated signal was observed in IFNAR^−/−^ mice following both IN and IP infection and in C57BL/6J mice following IP infection. Strain-dependent differences were also evident, with slower signal decline and more persistent head signal observed following NiV-B infection compared to NiV-M. Increases in signal within the head or chest frequently preceded the onset of neurological or respiratory manifestations, respectively, supporting a temporal link between viral burden and clinical outcome. These findings extend prior imaging studies in IFNAR^−/−^ mice using firefly-luciferase-expressing NiV-M [9] and rCedV expressing NiV-B F and G proteins [11], demonstrating sustained dissemination and delayed resolution beyond early acute time points.

There are inherent limitations to this experimental system that should be considered when interpreting these findings. Imaging signal reflects reporter-based viral activity and does not alone establish the presence of infectious virus. As the intracellular half-life of BREP has not been defined, persistent reporter signal may reflect accumulated protein rather than ongoing viral replication. Inclusion of a destabilization sequence, such as a PEST motif, could reduce signal retention. However, the near-complete clearance of reporter activity from immunocompetent C57BL/6J mice infected with rNiV-BREP constructs suggests that persistent reporter signal is not solely attributable to reporter stability. In addition, IFNAR^−^/^−^ mice may not fully recapitulate persistence dynamics in immunocompetent hosts. Finally, consistent with other reporter systems [29–32], incorporation of the reporter was associated with mild attenuation relative to wild-type virus, which may influence disease kinetics while also enabling longitudinal assessment of survival and persistence. Nevertheless, concordance between longitudinal imaging, RT-qPCR, and ISH supports the biological relevance of the persistence patterns observed here. Together, these data support a model of spatially restricted NiV persistence and incomplete clearance following apparent recovery, provide biological context for understanding relapsing neurological disease, and establish an in vivo framework for future studies to define mechanisms of persistence and evaluate strategies to enhance viral resolution in restricted tissue compartments.

## Supplementary Material

Supplement_NiVImaging_EMI_resub.pdf

## Data Availability

The datasets generated and/or analyzed during the current study are available from the corresponding author upon reasonable request.
