# Supplementary material for: Longitudinal in vivo imaging reveals asynchronous, incomplete Nipah virus clearance with prolonged focal CNS involvement in IFNAR−/− mice
Source: Emerg Microbes Infect. 2026 Jul 24;15(1):2703394. doi: 10.1080/22221751.2026.2703394 (PMC13403577; doi:10.1080/22221751.2026.2703394)
Supplement: Supplement_NiVImaging_EMI_resub.pdf [file TEMI_A_2703394_SM5941.pdf]

Supplementary Data

Longitudinal in vivo imaging reveals asynchronous, incomplete Nipah virus clearance with prolonged focal CNS involvement in IFNAR$^{-/-}$ mice

Katherine A. Davies[1,2], Stephen R. Welch[3], JoAnn D. Coleman-McCray[3], Georgia Ficarra[4], Jana M. Ritter[4], Teresa E. Sorvillo[1], Virginia Aida-Ficken[3,5], Shilpi Jain[3], César G. Albariño[3], Joel M. Montgomery[3], Michael K. Lo[3], Christina F. Spiropoulou[3], Jessica R. Spengler[3*]

[1]CDC Foundation assigned to Viral Special Pathogens Branch, Division of High-Consequence Pathogens and Pathology, Centers for Disease Control and Prevention, Atlanta, GA, USA.
[2]Zoonotic and Emerging Disease Research Unit, National Bio and Agro-Defense Facility, Agricultural Research Service, United States Department of Agriculture, Manhattan, Kansas, USA
[3]Viral Special Pathogens Branch, Division of High-Consequence Pathogens and Pathology, Centers for Disease Control and Prevention, Atlanta, Georgia, USA
[4]Infectious Diseases Pathology Branch, Division of High-Consequence Pathogens and Pathology, Centers for Disease Control and Prevention, Atlanta, GA, USA.
[5]Foreign Animal Disease Diagnostic Laboratory, National Veterinary Services Laboratories, National Bio and Agro-Defense Facility, United States Department of Agriculture, Manhattan, KS, USA.

*Corresponding author: Jessica R. Spengler; wsk7@cdc.gov

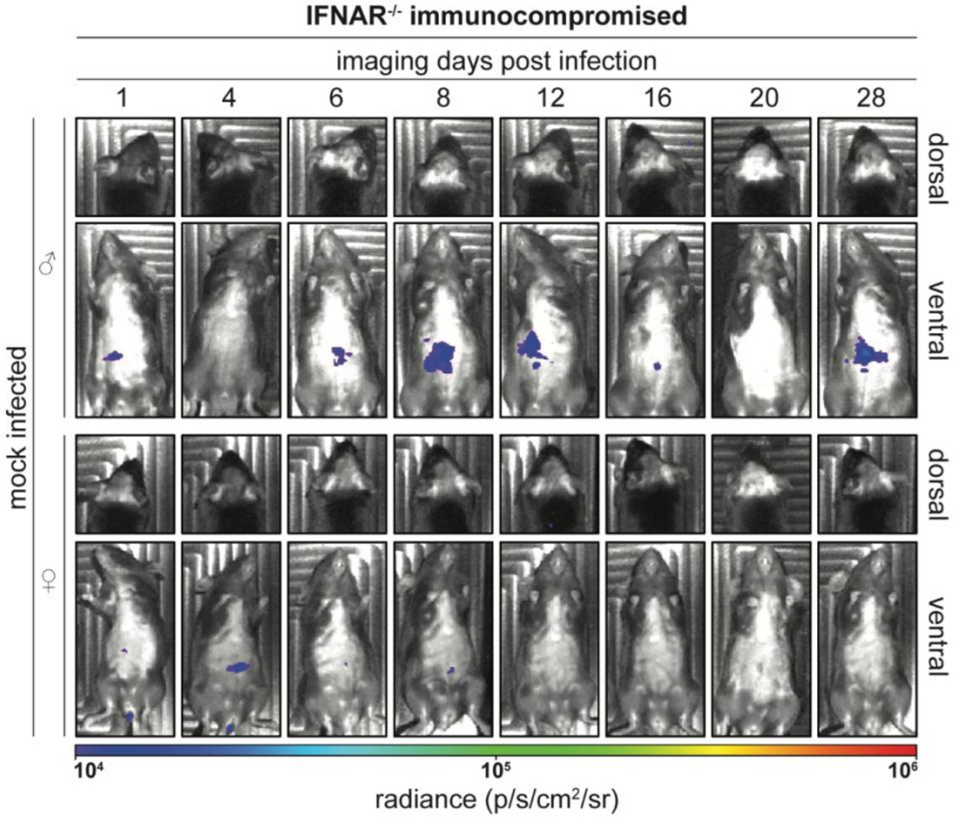

Figure S1. In vivo imaging of mock-infected IFNAR$^{-/-}$ mice reveals minimal background signal. Mock-infected IFNAR$^{-/-}$ mice were imaged under conditions identical to mice infected with reporter-expressing Nipah virus strains to assess background signal and artifact presence. Representative images of male (♂) and female (♀) mice are shown. Radiance (p/s/cm²/sr) is indicated by the scale bar.

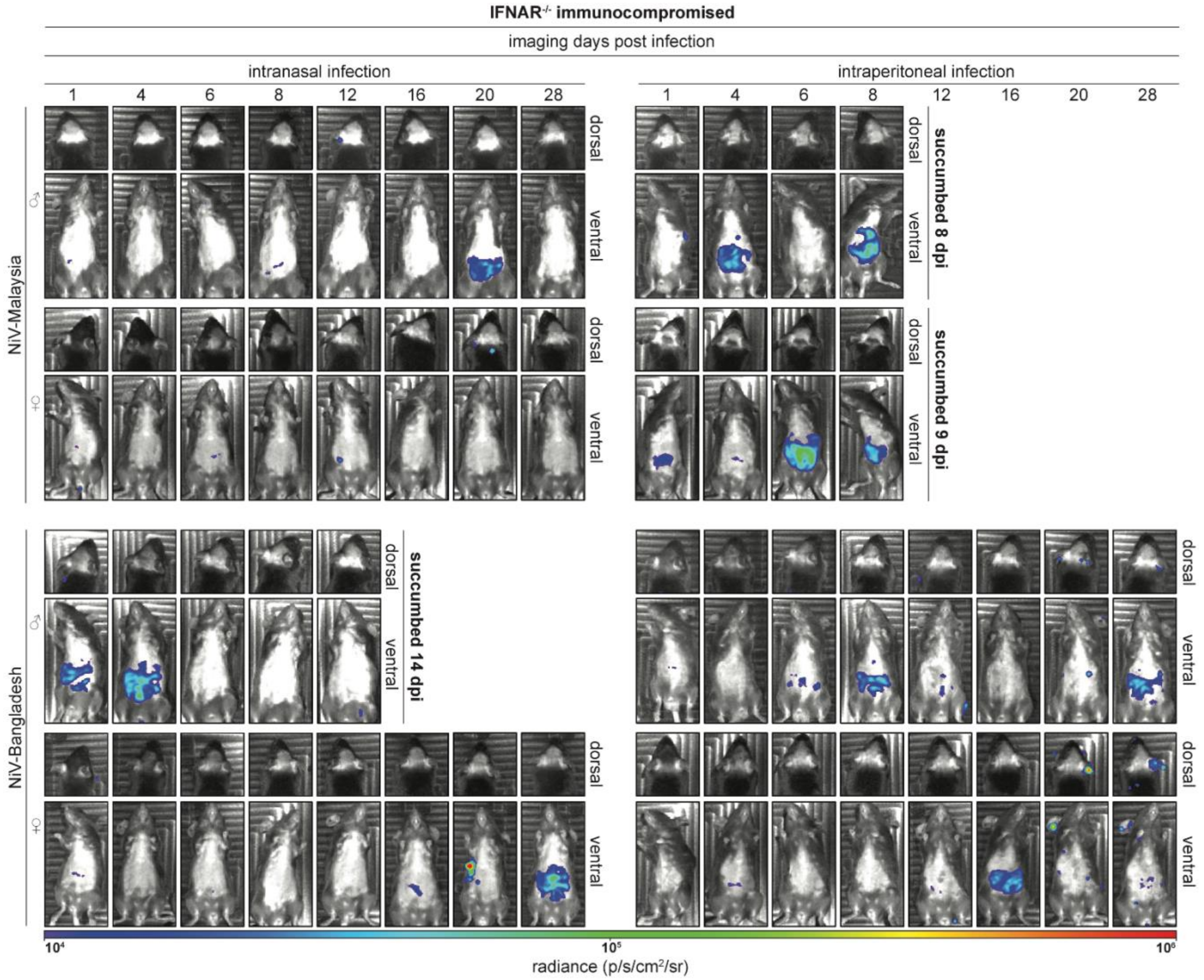

Figure S2. In vivo imaging of IFNAR$^{-/-}$ mice infected with wild-type Nipah virus reveals minimal background signal. Nipah virus Malaysia (NiV-Malaysia) or Nipah virus Bangladesh (NiV-Bangladesh) infected IFNAR$^{-/-}$ mice were imaged under conditions identical to those used for reporter-expressing NiV strains to assess background signal and artifact presence. Representative images of male (♂) and female (♀) mice are shown. Radiance (p/s/cm²/sr) is indicated by the scale bar.

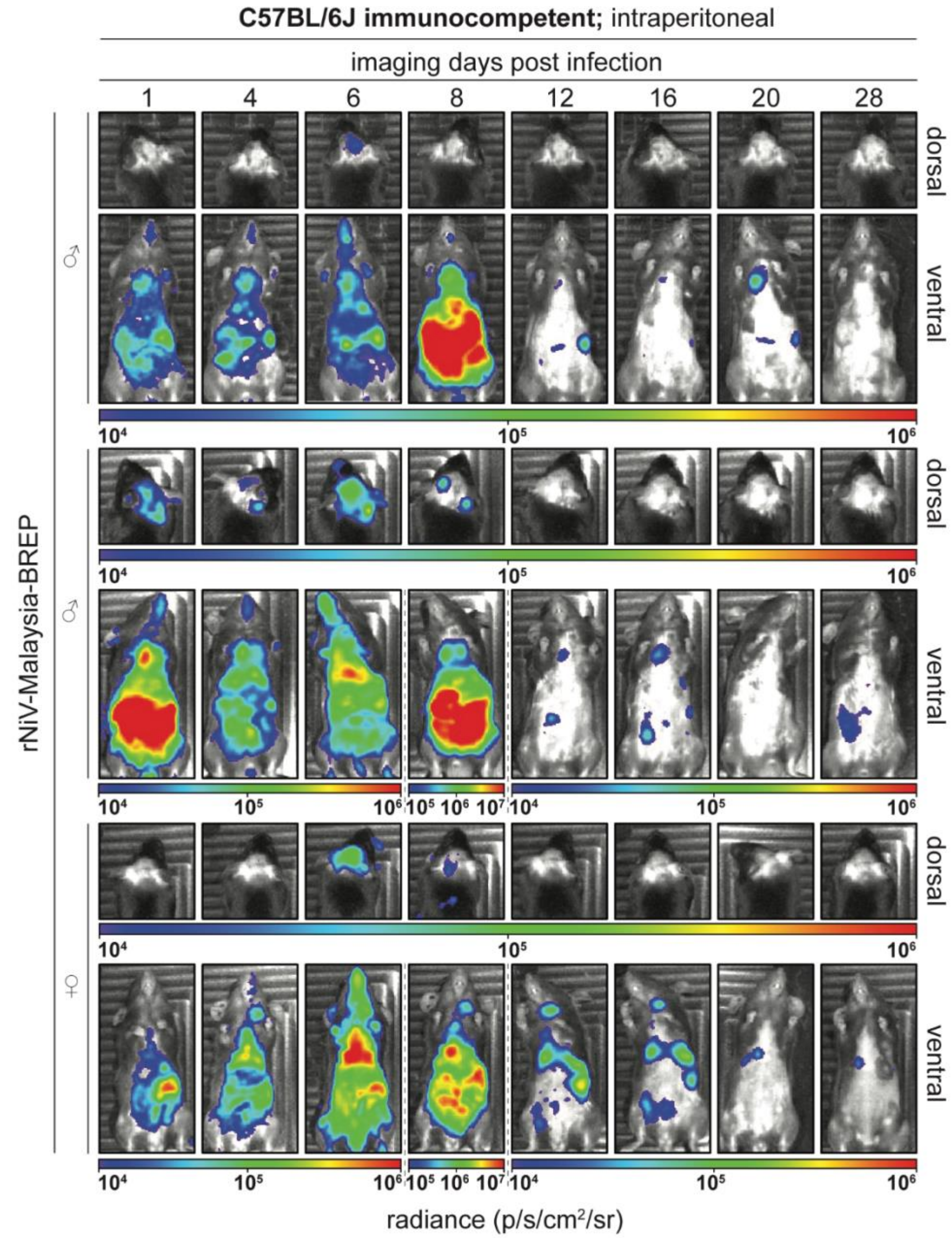

Figure S3. In vivo imaging of immunocompetent mice infected with rNiV-M-BREP reveals widespread virus dissemination. C57BL/6J mice (n=5/group) were infected intraperitoneally with 10^6 $TCID_{50}$ recombinant NiV-M expressing BREP (rNiV-Malaysia-BREP) and imaged at 1, 4, 6, 8, 12, 16, 20, and 28 days post infection. Radiance (p/s/cm²/sr) is indicated by the scale bar. The dashed vertical line between imaging panels indicates a change in radiance scale. Images are representative of mice not included in main text figures.

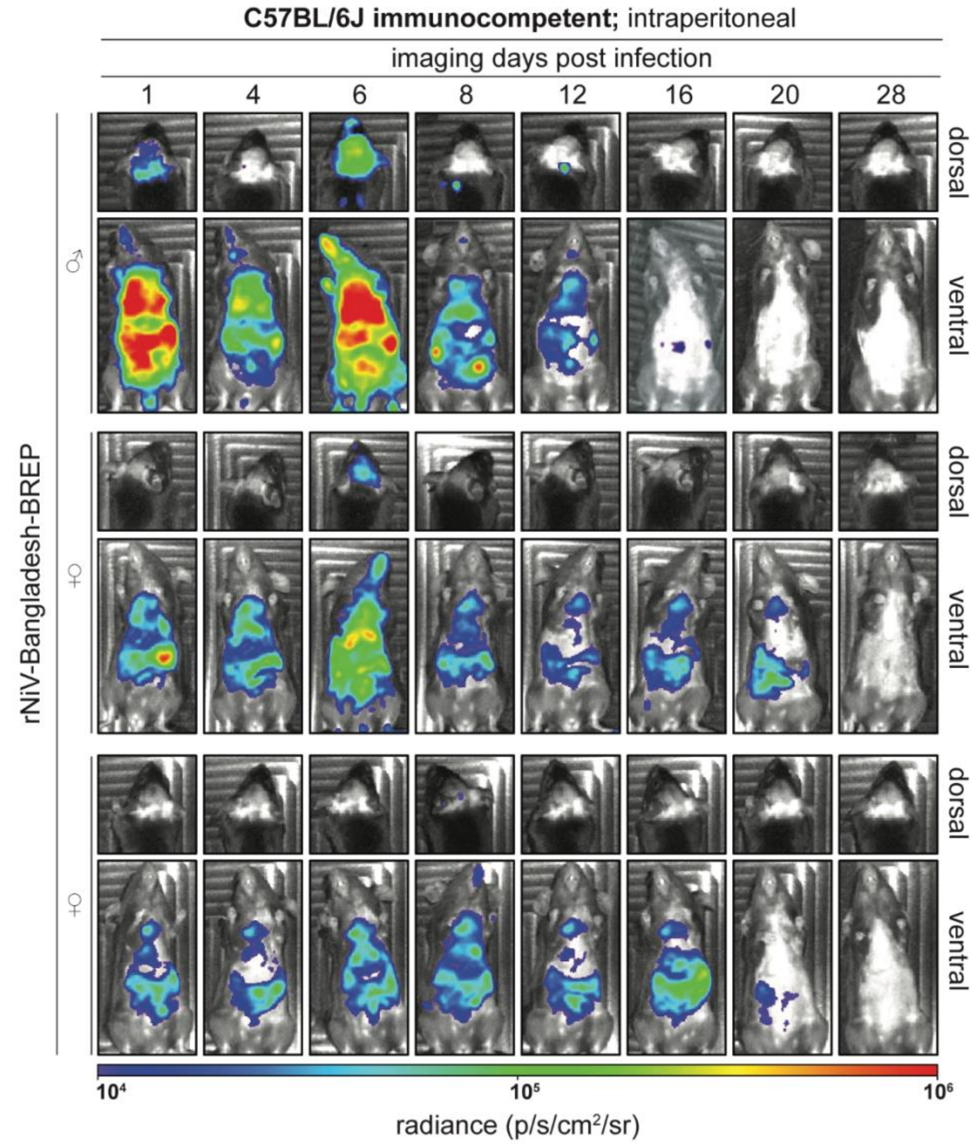

Figure S4. In vivo imaging of immunocompetent mice infected with rNiV-B-BREP reveals widespread virus dissemination. C57BL/6J mice (n=5/group) were infected intraperitoneally with 10^6 $TCID_{50}$ recombinant NiV-B expressing BREP (rNiV-Bangladesh-BREP) and imaged at 1, 4, 6, 8, 12, 16, 20, and 28 days post infection. Radiance (p/s/cm²/sr) is indicated by the scale bar. Images are representative of mice not included in main text figures.

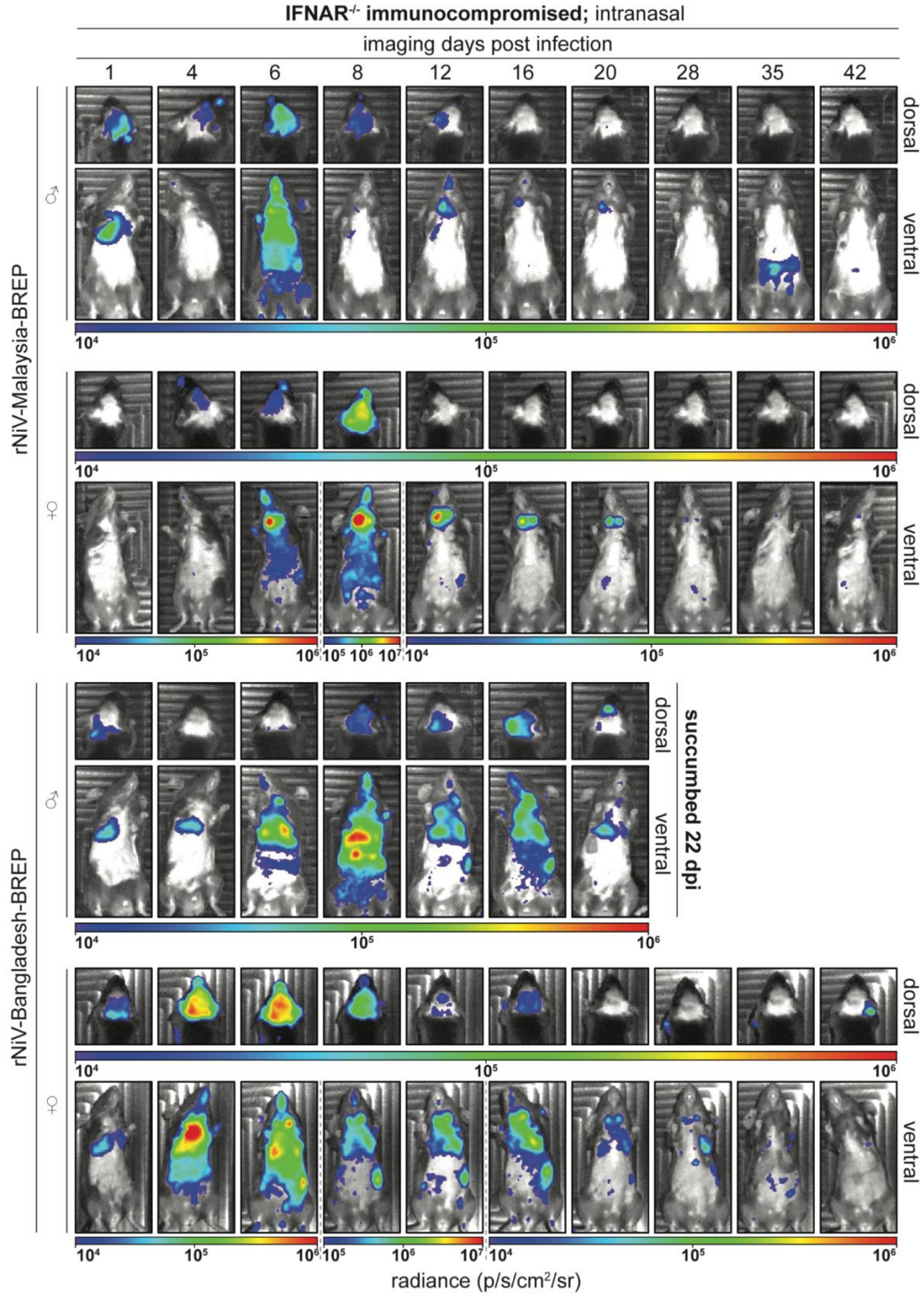

IFNAR-/- immunocompromised; intranasal
imaging days post infection
1
4
6
8
12
16
20
28
35
42
dorsal
ventral
rNiV-Malaysia-BREP
♂
♀
rNiV-Bangladesh-BREP
succumbed 22 dpi
10⁴
10⁵
10⁶
10⁷
radiance (p/s/cm²/sr)

Figure S5. In vivo imaging of IFNAR$^{-/-}$ mice infected intranasally with Nipah virus results in upper body dissemination and neuroinvasion. IFNAR$^{-/-}$ mice were infected intranasally with 10^6 $TCID_{50}$ recombinant NiV-M expressing BREP (rNiV-Malaysia-BREP) or recombinant NiV-B expressing BREP (rNiV-Bangladesh-BREP) and imaged at 1, 4, 6, 8, 12, 16, 20, 28, 35, and 42 days post infection. Radiance (p/s/cm²/sr) is indicated by the scale bar. The dashed vertical line between imaging panels indicates a change in radiance scale. Images are representative of mice not included in main text figures.

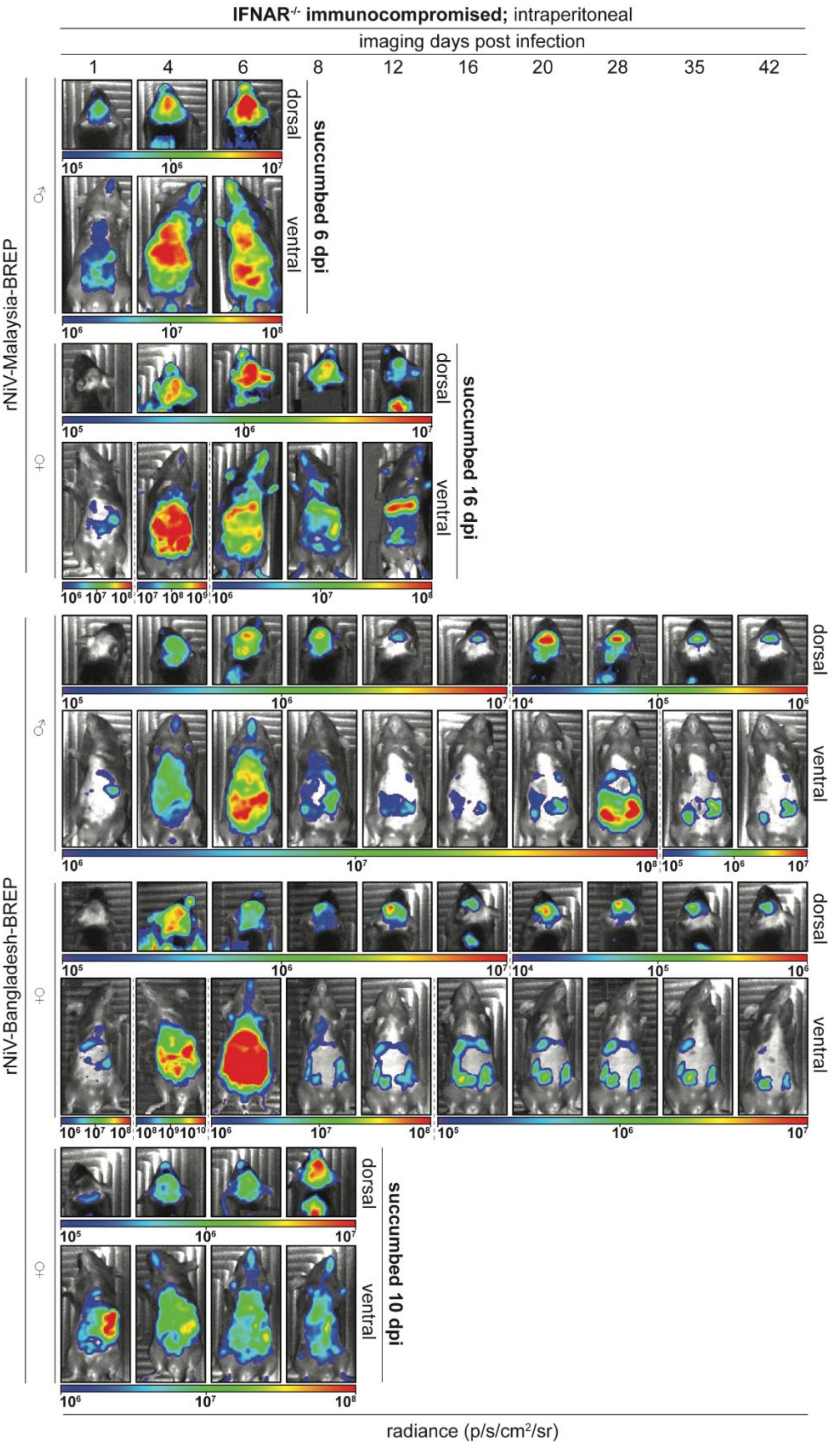

IFNAR-/- immunocompromised; intraperitoneal
imaging days post infection
1 4 6 8 12 16 20 28 35 42
rNiV-Malaysia-BREP
♂
♀
dorsal
ventral
succumbed 6 dpi
succumbed 16 dpi
rNiV-Bangladesh-BREP
succumbed 10 dpi
radiance (p/s/cm2/sr)

Figure S6. In vivo imaging of IFNAR$^{-/-}$ mice infected intraperitoneally with Nipah virus results in widespread dissemination and sites of persistent reporter-associated signal. IFNAR$^{-/-}$ mice were infected intraperitoneally with 10^6 $TCID_{50}$ recombinant NiV-M expressing BREP (rNiV-Malaysia-BREP) or recombinant NiV-B expressing BREP (rNiV-Bangladesh-BREP) and imaged at 1, 4, 6, 8, 12, 16, 20, 28, 35, and 42 days post infection. Radiance (p/s/cm²/sr) is indicated by the scale bar. The dashed vertical line between imaging panels indicates a change in radiance scale. Images are representative of mice not included in main text figures.

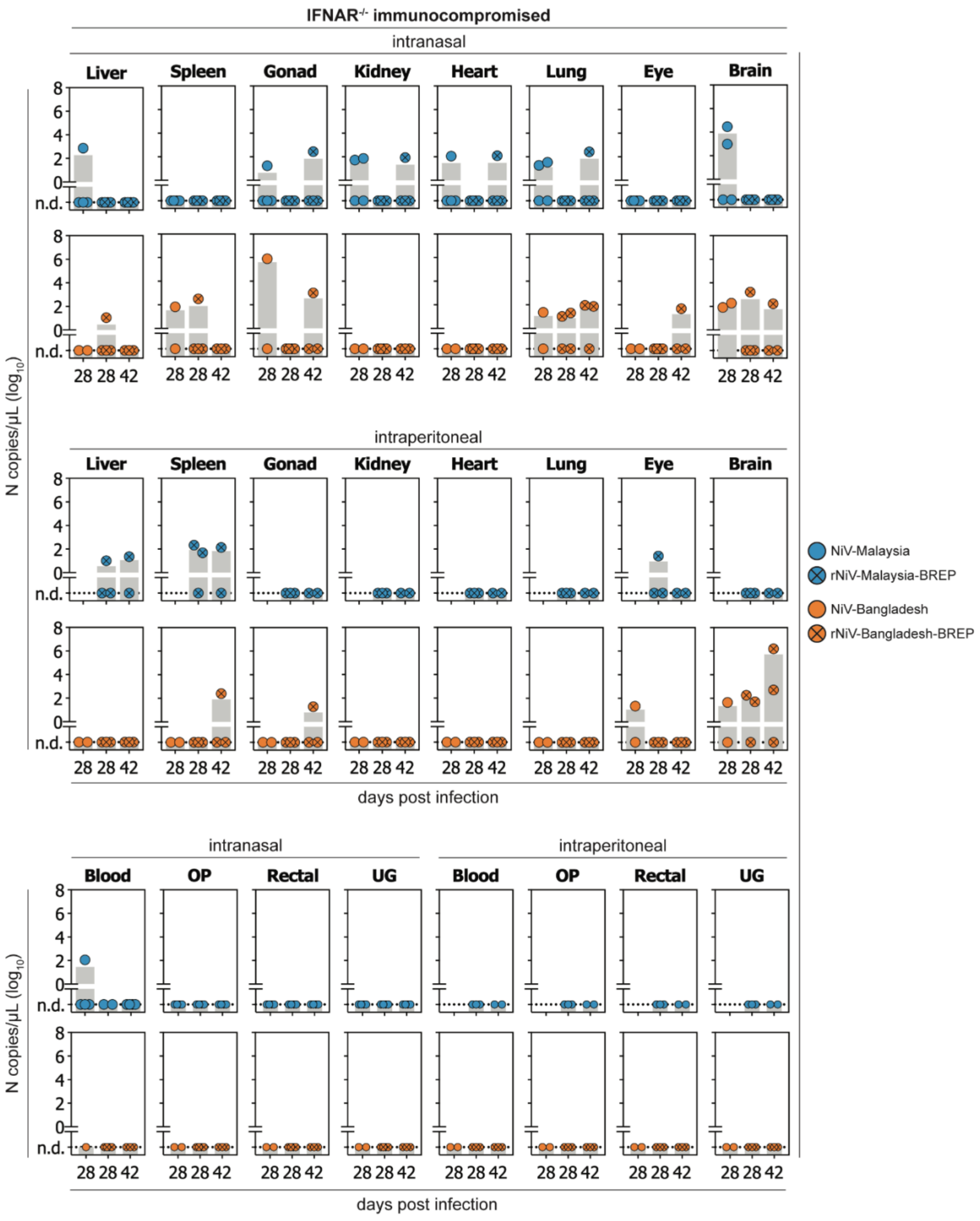
IFNAR-/- immunocompromised
intranasal
Liver
Spleen
Gonad
Kidney
Heart
Lung
Eye
Brain
intraperitoneal
N copies/µL (log10)
n.d.
28 28 42
days post infection
Blood
OP
Rectal
UG
NiV-Malaysia
rNiV-Malaysia-BREP
NiV-Bangladesh
rNiV-Bangladesh-BREP

Figure S7. Detection of viral RNA in tissues by RT-qPCR after clinical recovery reveals rare RNA persistence in distinct anatomical sites. IFNAR$^{-/-}$ mice were infected intranasally or intraperitoneally with 10^6 $TCID_{50}$ NiV-Malaysia (NiV-M), recombinant NiV-M expressing BREP (rNiV-Malaysia-BREP), NiV-Bangladesh (NiV-B), or recombinant NiV-B expressing BREP (rNiV-Bangladesh-BREP). NiV-M- or NiV-B-infected mice were euthanized at 28 days post-infection (dpi), and rNiV-Malaysia-BREP- or rNiV-Bangladesh-BREP-infected mice were euthanized at 28 or 42 dpi. Tissue (liver, spleen, gonad [testis, ovary], kidney, heart, lung, eye, and brain), blood, and mucosal swabs (oropharyngeal [OP], rectal, and urogenital [UG]) were collected. RNA was extracted and viral RNA loads (nucleoprotein [N] gene copies per µL) were quantified by RT-qPCR. Each data point represents an individual animal, the grey bar indicates the group mean. 'n.d.' no RNA was detected.

Table S1. Individual mouse infection conditions, imaging timepoints, and clinical outcomes following Nipah virus infection.

Animal ID	Mouse strain	Virus Strain	Reporter	Route	Imaging timepoint [dpi]	Euthanized [dpi]	Max Clinical Score
3205-4-1	C57BL/6J	NiV-M	BREP	IP	0, 1, 4, 6, 8, 12, 16, 20, 28	28	0
3205-4-2	C57BL/6J	NiV-M	BREP	IP	0, 1, 4, 6, 8, 12, 16, 20, 28	28	0
3205-4-3	C57BL/6J	NiV-M	BREP	IP	0, 1, 4, 6, 8, 12, 16, 20, 28	28	0
3205-4-4	C57BL/6J	NiV-M	BREP	IP	0, 1, 4, 6, 8, 12, 16, 20, 28	28	0
3205-4-5	C57BL/6J	NiV-M	BREP	IP	0, 1, 4, 6, 8, 12, 16, 20, 28	28	0
3205-4-6	C57BL/6J	NiV-B	BREP	IP	0, 1, 4, 6, 8, 12, 16, 20, 28	28	0
3205-4-7	C57BL/6J	NiV-B	BREP	IP	0, 1, 4, 6, 8, 12, 16, 20, 28	28	0
3205-4-8	C57BL/6J	NiV-B	BREP	IP	0, 1, 4, 6, 8, 12, 16, 20, 28	28	0
3205-4-9	C57BL/6J	NiV-B	BREP	IP	0, 1, 4, 6, 8, 12, 16, 20, 28	28	0
3205-4-10	C57BL/6J	NiV-B	BREP	IP	0, 1, 4, 6, 8, 12, 16, 20, 28	28	0
3205-3-1	IFNAR$^{-/-}$	NiV-M	BREP	IN	0, 1, 4, 6, 8, 12, 16, 20, 28, 35, 42	42	0
3205-3-2	IFNAR$^{-/-}$	NiV-M	BREP	IN	0, 1, 4, 6, 8, 12, 16, 20, 28, 35, 42	42	0
3205-3-3	IFNAR$^{-/-}$	NiV-M	BREP	IN	0, 1, 4, 6, 8, 12, 16, 20, 28, 35, 42	42	0
3205-3-4	IFNAR$^{-/-}$	NiV-M	BREP	IN	0, 1, 4, 6, 8, 12, 16, 20, 28, 35, 42	42	0
3205-3-5	IFNAR$^{-/-}$	NiV-M	BREP	IP	0, 1, 4, 6, 8, 12, 16, 20, 28, 35, 42	42	10
3205-3-6	IFNAR$^{-/-}$	NiV-M	BREP	IP	0, 1, 4, 6	6	10
3205-3-7	IFNAR$^{-/-}$	NiV-M	BREP	IP	0, 1, 4, 6, 8, 12, 16	16	0
3205-3-8	IFNAR$^{-/-}$	NiV-M	BREP	IP	0, 1, 4, 6, 8, 12	15	10
3205-3-9	IFNAR$^{-/-}$	NiV-M	None	IN	0, 1, 4, 6, 8, 12, 16, 20, 28	28	2
3205-3-10	IFNAR$^{-/-}$	NiV-M	None	IN	0, 1, 4, 6, 8, 12, 16, 20, 28	28	0
3205-3-11	IFNAR$^{-/-}$	NiV-M	None	IN	0, 1, 4, 6, 8, 12, 16, 20, 28	28	5
3205-3-12	IFNAR$^{-/-}$	NiV-M	None	IN	0, 1, 4, 6, 8, 12, 16, 20, 28	28	0
3205-3-13	IFNAR$^{-/-}$	NiV-M	None	IP	0, 1, 4, 6	6	10
3205-3-14	IFNAR$^{-/-}$	NiV-M	None	IP	0, 1, 4, 6, 8	8	10
3205-3-15	IFNAR$^{-/-}$	NiV-M	None	IP	0, 1, 4, 6, 8	8	10
3205-3-16	IFNAR$^{-/-}$	NiV-M	None	IP	0, 1, 4, 6, 8	9	10
3205-3-17	IFNAR$^{-/-}$	NiV-B	BREP	IN	0, 1, 4, 6, 8, 12, 16, 20, 28, 35, 42	42	0
3205-3-18	IFNAR$^{-/-}$	NiV-B	BREP	IN	0, 1, 4, 6, 8, 12, 16, 20	26	10
3205-3-19	IFNAR$^{-/-}$	NiV-B	BREP	IN	0, 1, 4, 6, 8, 12, 16, 20, 28, 35, 42	42	0
3205-3-20	IFNAR$^{-/-}$	NiV-B	BREP	IN	0, 1, 4, 6, 8, 12, 16, 20, 28, 35, 42	42	0
3205-3-21	IFNAR$^{-/-}$	NiV-B	BREP	IP	0, 1, 4, 6, 8, 12, 16, 20, 28, 35, 42	42	0
3205-3-22	IFNAR$^{-/-}$	NiV-B	BREP	IP	0, 1, 4, 6, 8, 12, 16, 20, 28, 35, 42	42	0
3205-3-23	IFNAR$^{-/-}$	NiV-B	BREP	IP	0, 1, 4, 6, 8, 12, 16, 20, 28, 35, 42	42	5
3205-3-24	IFNAR$^{-/-}$	NiV-B	BREP	IP	0, 1, 4, 6, 8	10	10
3205-3-25	IFNAR$^{-/-}$	NiV-B	None	IN	0, 1, 4, 6, 8, 12	14	10
3205-3-26	IFNAR$^{-/-}$	NiV-B	None	IN	0, 1, 4, 6, 8, 12	15	10
3205-3-27	IFNAR$^{-/-}$	NiV-B	None	IN	0, 1, 4, 6, 8, 12, 16, 20, 28	28	7
3205-3-28	IFNAR$^{-/-}$	NiV-B	None	IN	0, 1, 4, 6, 8, 12, 16, 20, 28	28	0
3205-3-29	IFNAR$^{-/-}$	NiV-B	None	IP	0, 1, 4, 6	7	10

Animal ID	Mouse strain	Virus Strain	Reporter	Route	Imaging timepoint [dpi]	Euthanized [dpi]	Max Clinical Score
3205-3-30	$IFNAR^{-/-}$	NiV-B	None	IP	0, 1, 4, 6, 8, 12, 16, 20, 28	28	0
3205-3-31	$IFNAR^{-/-}$	NiV-B	None	IP	0, 1, 4, 6, 8	8	10
3205-3-32	$IFNAR^{-/-}$	NiV-B	None	IP	0, 1, 4, 6, 8, 12, 16, 20, 28	28	0
3205-3-33	$IFNAR^{-/-}$	None	None	IP	0, 1, 4, 6, 8, 12, 16, 20, 28	28	0
3205-3-34	$IFNAR^{-/-}$	None	None	IP	0, 1, 4, 6, 8, 12, 16, 20, 28	28	0
3205-3-35	$IFNAR^{-/-}$	None	None	IP	0, 1, 4, 6, 8, 12, 16, 20, 28	28	0
3205-3-36	$IFNAR^{-/-}$	None	None	IP	0, 1, 4, 6, 8, 12, 16, 20, 28	28	0
3205-3-37	$IFNAR^{-/-}$	NiV-M	BREP	IN	None	28	0
3205-3-38	$IFNAR^{-/-}$	NiV-M	BREP	IN	None	28	0
3205-3-39	$IFNAR^{-/-}$	NiV-M	BREP	IN	None	28	0
3205-3-40	$IFNAR^{-/-}$	NiV-M	BREP	IN	None	28	0
3205-3-41	$IFNAR^{-/-}$	NiV-M	BREP	IP	None	28	0
3205-3-42	$IFNAR^{-/-}$	NiV-M	BREP	IP	28, 35, 42	42	5
3205-3-43	$IFNAR^{-/-}$	NiV-M	BREP	IP	None	28	2
3205-3-44	$IFNAR^{-/-}$	NiV-M	BREP	IP	None	28	0
3205-3-45	$IFNAR^{-/-}$	NiV-B	BREP	IN	None	28	0
3205-3-46	$IFNAR^{-/-}$	NiV-B	BREP	IN	None	28	0
3205-3-47	$IFNAR^{-/-}$	NiV-B	BREP	IN	None	28	0
3205-3-48	$IFNAR^{-/-}$	NiV-B	BREP	IN	None	28	0
3205-3-49	$IFNAR^{-/-}$	NiV-B	BREP	IP	None	28	0
3205-3-50	$IFNAR^{-/-}$	NiV-B	BREP	IP	None	28	2
3205-3-51	$IFNAR^{-/-}$	NiV-B	BREP	IP	None	8	10
3205-3-52	$IFNAR^{-/-}$	NiV-B	BREP	IP	None	28	5

Abbreviations: C57BL/6J, immunocompetent mice; $IFNAR^{-/-}$, type I interferon receptor knockout mice; NiV-M, Nipah virus Malaysia; NiV-B, Nipah virus Bangladesh; BREP, recombinant reporter virus expressing a bioluminescent red protein construct which comprises a red-shifted NanoLuciferase (teLuc) linked to mScarlet-1; IN, intranasal; IP, intraperitoneal; dpi, days post-infection. Mice were infected via the IN route ($IFNAR^{-/-}$ mice only) or IP route at 10^6 $TCID_{50}$. Imaging timepoints shown for each animal indicate dpi. Clinical scores ranged from 0 to 10, with 10 indicating endpoint criteria were reached.
